# Running the risk: extreme right ventricular remodelling in an endurance athlete—a case report

**DOI:** 10.1093/ehjcr/ytaf598

**Published:** 2025-11-20

**Authors:** Jonathan S Ahn, Elizabeth H Dineen, Kristina H Haugaa, Bassam Yaghmour, Jennifer Xu

**Affiliations:** Department of Medicine, Loma Linda University, 11234 Anderson St., Room 1503, Loma Linda, CA 92354, USA; Department of Cardiovascular Medicine, Mayo Clinic Florida, 4500 San Pablo Road, Jacksonville, FL 32224, USA; ProCardio Center for Innovation, Department of Cardiology, Oslo University Hospital, Postboks 4950 Nydalen, 0424 Oslo, Norway; Division of Pulmonary Diseases & Critical Care Medicine, Department of Medicine, University of California, Irvine, 333 City Blvd. West, Suite 400, Orange, CA, USA; Division of Cardiology, Department of Medicine, University of California, 333 City Blvd. West, Suite 400, Orange, CA, USA

**Keywords:** Cardiac remodelling, Athletic heart, Right ventricular dilation, Exercise-induced cardiomyopathy, Case report

## Abstract

**Background:**

Distinguishing physiological cardiac adaptation from pathological remodelling in endurance athletes is challenging. While the athletic heart typically demonstrates balanced biventricular enlargement, extreme exercise may rarely trigger disproportionate right ventricular (RV) changes that challenge diagnostic classification.

**Case summary:**

A 47-year-old male ultra-endurance athlete presented with exertional symptoms 3 months after recovery from a pulmonary embolism (PE). Initial post-PE echocardiography was normal. After resuming extreme training, he developed severe RV dilation (basal diameter 5.7 cm on echocardiography; RV volume index 92 cc/m² on cardiac magnetic resonance imaging) disproportionate to the left ventricle, with preserved systolic function and no late gadolinium enhancement. Cardiopulmonary exercise testing revealed significant chronotropic incompetence with below-expected peak oxygen uptake for his elite training status. Symptoms and ventricular ectopy improved with detraining, but severe RV dilation persisted. Evaluation excluded arrhythmogenic cardiomyopathy and significant pulmonary vascular sequelae.

**Discussion:**

This case illustrates disproportionate RV remodelling precipitated by the resumption of extreme exercise in a susceptible athlete post-PE. The temporal dissociation from the PE and absence of residual clot burden implicate exercise-related haemodynamic stress as the primary driver, challenging the boundary between physiological adaptation and maladaptation. These findings highlight a potential maladaptive response to extreme exercise, possibly potentiated by a prior vascular insult, and underscore the value of integrated functional assessment and monitored detraining in athletes with borderline findings.

Learning points

- Extreme exercise can trigger disproportionate and persistent right ventricular dilation beyond normal athletic physiologic norms.
- Monitored detraining can clarify diagnosis and aid recovery to help determine surveillance needs and timing of safe return to training.

## Introduction

Distinguishing extreme physiologic adaptation from early pathologic remodelling in endurance athletes remains a challenge in sports cardiology. While the athletic heart typically exhibits balanced biventricular enlargement, extreme exercise training may induce disproportionate right ventricular (RV) changes. In this report, we present a case of rapid-onset, persistent severe RV dilation in an ultra-endurance athlete.

## Summary figure

**Figure:**
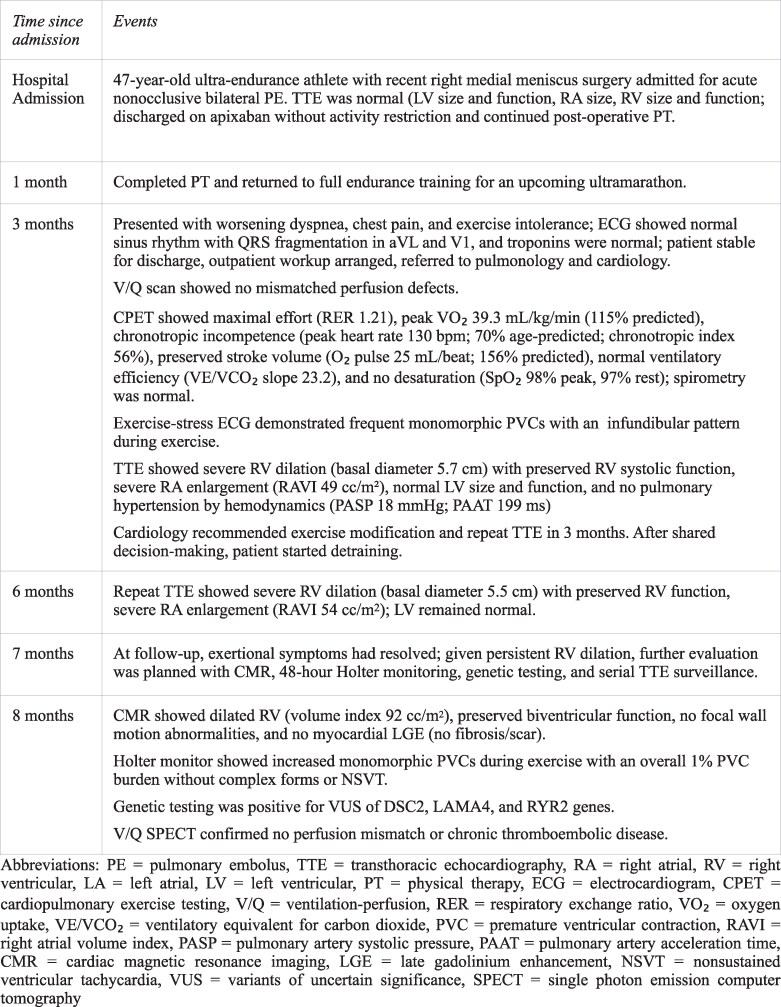


## Case presentation

**Figure 1:**
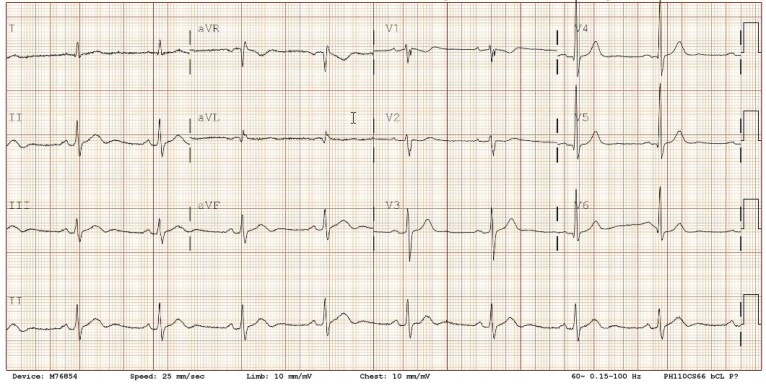
The electrocardiogram shows normal sinus rhythm with QRS fragmentation in aVL and V1, normal early repolarization patterns, and no epsilon waves.

A 47-year-old male ultra-endurance athlete (41-year training age) presented with exertional dyspnoea, chest pain, and exercise intolerance 3 months after a nonocclusive pulmonary embolism (PE). Vitals included blood pressure of 132/77 mmHg and a heart rate of 51 beats/min. Physical exam revealed clear lungs and no murmurs, jugular venous distention, or oedema. An electrocardiogram (ECG) showed normal sinus rhythm with QRS fragmentation in aVL and V1, normal early repolarization patterns, and no epsilon waves (*Figure 1*).

Past medical history was remarkable for a right medial meniscus repair surgery complicated by the postsurgical PE. Before injury, he trained 4–6 h daily by running, cycling, swimming, and weightlifting. A former Division I and Olympic soccer player, he has spent the past decade competing in ultramarathons worldwide. He denied illicit or performance-enhancing drug use. Family history included sudden cardiac death in an aunt and first cousin; no autopsies were performed.

At the time of PE, transthoracic echocardiogram (TTE) was unremarkable with a normal RV size and function. He was discharged on a factor Xa inhibitor and continued postoperative physical therapy. One month post-PE, he resumed training for a 52-mile ultramarathon. Two months later, he presented with exertional dyspnoea and chest pain. TTE revealed severe RV dilation (basal diameter 5.7 cm, proximal RV outflow tract diameter 4.5 cm) with severe right atrial enlargement (volume index 49 cc/m^2^, area 25.4 cm^2^) (see Supplementary material online, *Videos S1*-*S3*). RV systolic function was preserved (fractional area change 55%, tricuspid annular plane systolic excursion 2.97 cm, free wall global longitudinal strain −35%). The LV size and function were normal. Haemodynamics showed no evidence of pulmonary hypertension (estimated pulmonary artery systolic pressure 18 mmHg, acceleration time 199 ms).

**Figure 2:**
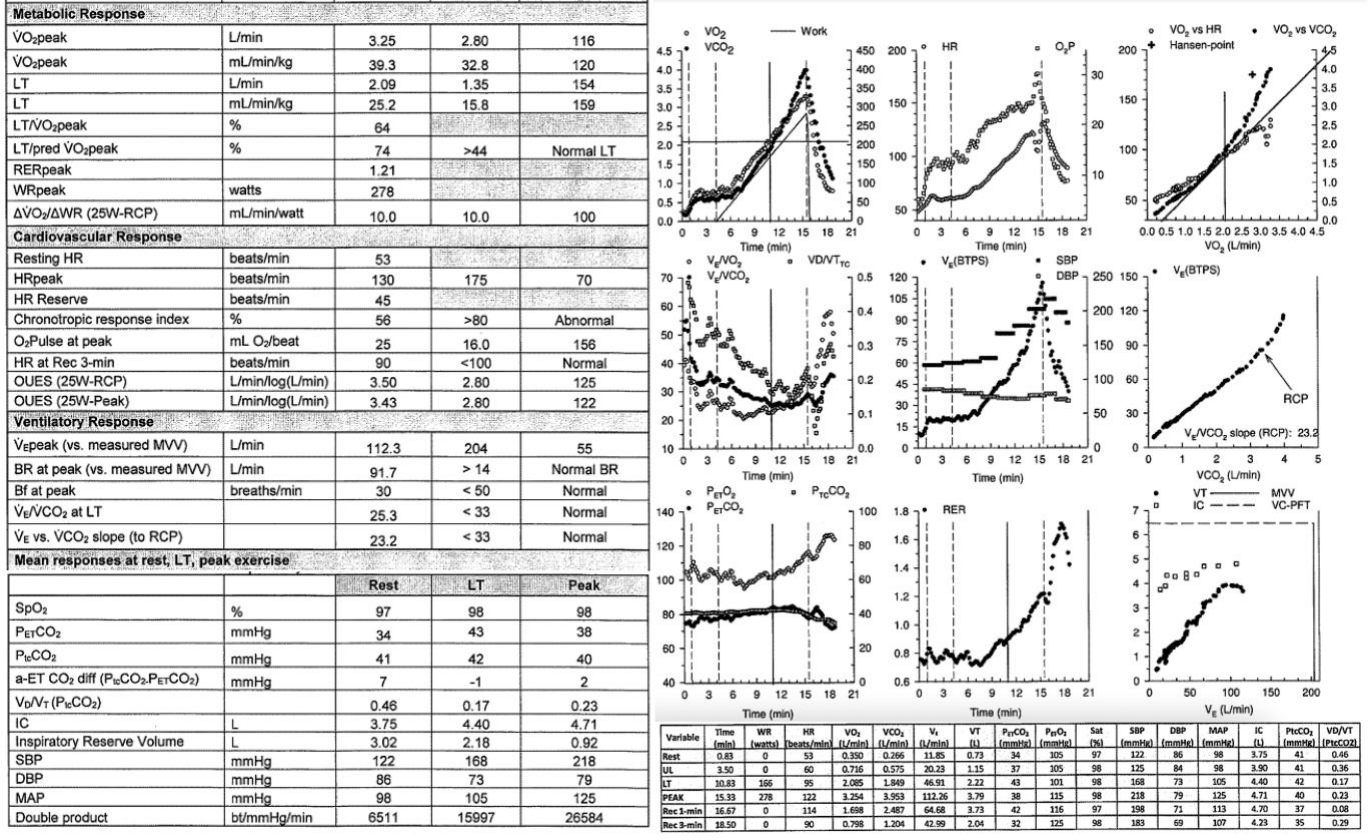
(Left) Key parameters of the cardiopulmonary exercise testing include maximal effort (peak RER 1.21), reduced chronotropic response (peak heart rate 130 bpm; 70% age-predicted), below-expected aerobic capacity for elite status (peak VO₂ 39.3 mL/kg/min; 115% predicted), preserved stroke volume (O₂ pulse 25 mL/beat; 156% predicted), normal ventilatory efficiency (VE/VCO₂ slope 23.2), and no desaturation (peak SpO₂ 98%); (Right) The nine-panel plot of the cardiopulmonary exercise testing.

**Figure 3:**
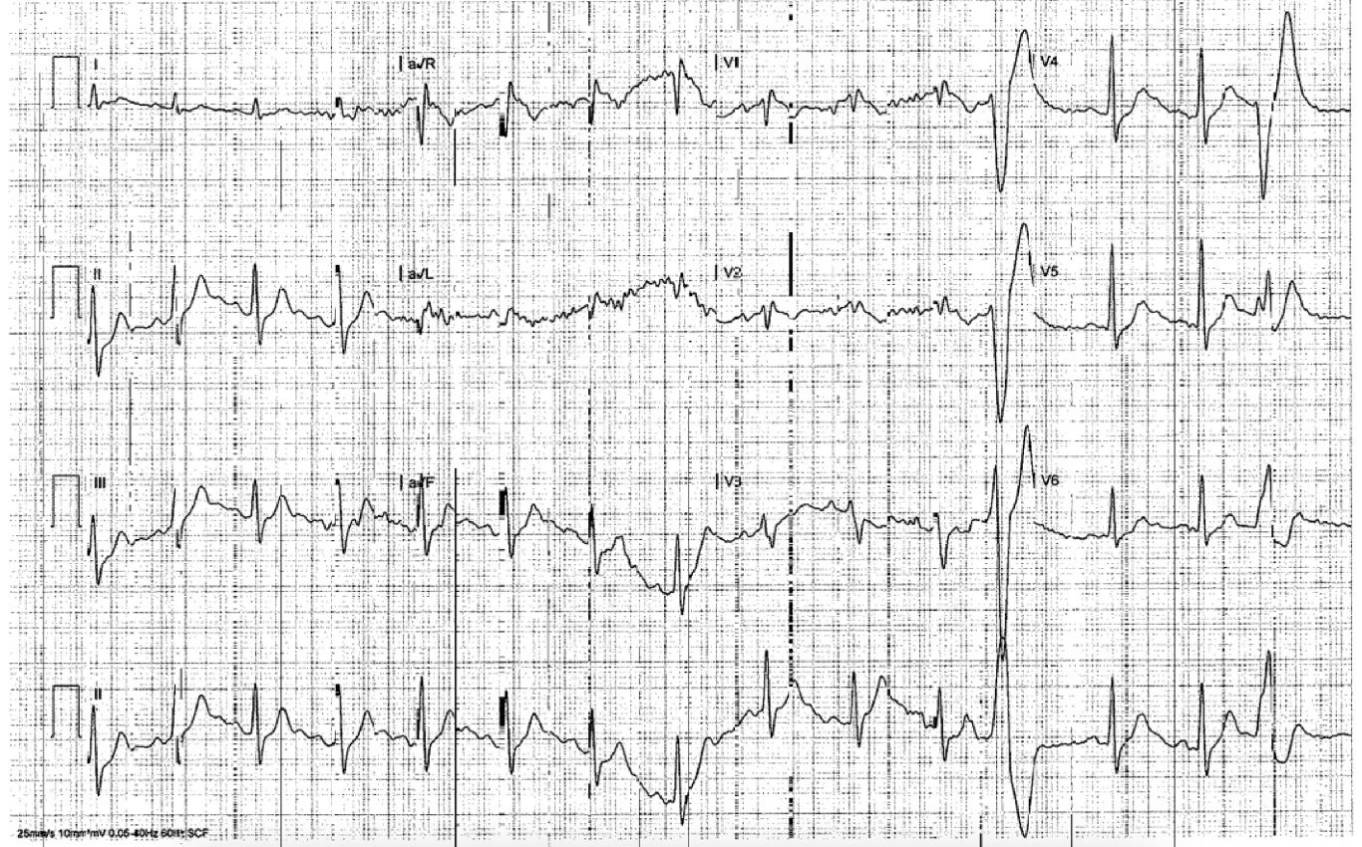
The stress electrocardiogram shows marginally increased QRS duration and premature ventricular contractions. Of note, the isolated premature ventricular contractions exhibit a LBBB morphology with inferior axis (infundibular) pattern.

Ventilation/perfusion (V/Q) scintigraphy was normal without mismatched defects. Cardiopulmonary exercise testing (CPET) (*Figure 2*) showed excellent effort [peak respiratory exchange ratio (RER) 1.21] with peak oxygen uptake (VO₂) of 3.25 L/min [39.3 mL/kg/min, 115% predicted based on the American College of Sports Medicine equation].^1^ He achieved a peak heart rate of 130 bpm (70% age-predicted maximum) with an abnormal chronotropic response index of 56%. Oxygen pulse was 25 mL/beat (156% predicted), indicating preserved stroke volume. Ventilatory efficiency was normal (VE/VCO₂ slope 23.2) with no desaturation (SpO₂ 98% peak, 97% at rest). Spirometry was normal [forced vital capacity (FVC) 6.46 L, forced expiratory volume in 1 s (FEV1) 5.22 L, FEV1/FVC 81%]. Arterial blood gas analysis was not obtained. During peak exercise, frequent monomorphic premature ventricular contractions (PVCs) of infundibular origin occurred (*Figure 3*).

**Figure 4:**
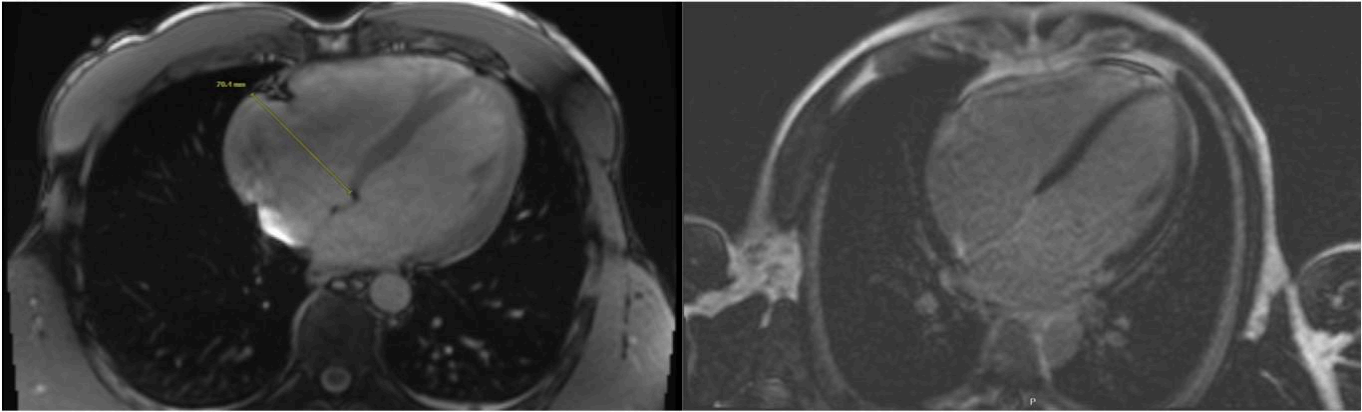
The cardiac magnetic resonance imaging shows: (left) still frame of an axial SSFP cine sequence, demonstrating a dilated right ventricle measuring around 70 mm at the base (volume index of 92 cc/m^2^) and without focal wall motion abnormalities to suggest arrhythmogenic right ventricular cardiomyopathy; (Right) Late gadolinium enhancement sequence, showing no signs of myocardial fibrosis.

**Figure 5:**
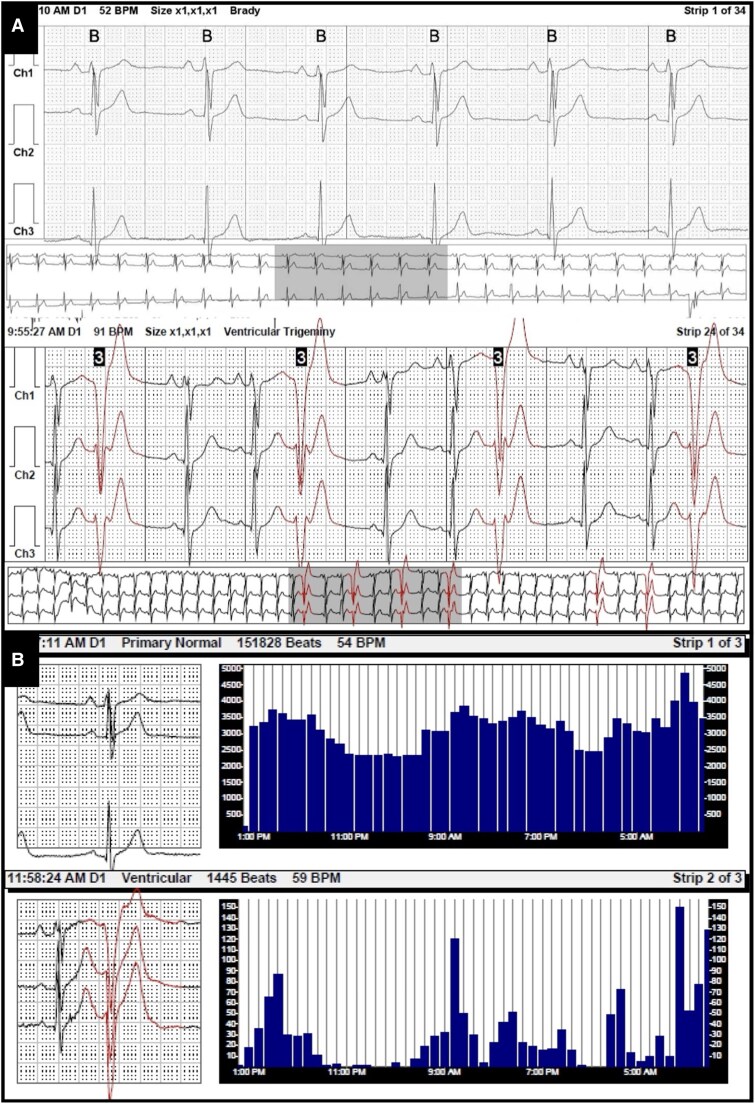
*A*) The 48-h three-channel Holter monitor shows increased frequency of monomorphic premature ventricular contractions of infundibular origin at elevated heart rates during training. *B*) Morphology review of the 48-h Holter shows an overall 1% PVC burden without nonsustained ventricular tachycardia. One couplet and zero triplets were noted.

Through shared decision-making with a sports cardiologist, he detrained and limited exercise to one hour daily, with plans to revisit testing and training. No pharmacologic therapy was initiated. Three months later, symptoms resolved but repeat TTE demonstrated persistent RV dilation (basal diameter 5.5 cm). Cardiac magnetic resonance imaging (CMR) showed an RV volume index of 92 cc/m^2^ without late gadolinium enhancement (LGE) or focal wall motion abnormalities (*Figure 4*). A 48-hour three-channel Holter monitor showed increased monomorphic PVCs during training (*Figure 5A*) with overall 1% PVC burden, with one couplet and zero triplets noted during monitoring (*Figure 5B*). V/Q single photon emission computer tomography (SPECT) confirmed no perfusion mismatch or chronic thromboembolic disease. Genetic testing found variants of uncertain significance in the DSC2, LAMA4, and RYR2 genes. He was referred to genetic counselling; family screening was recommended.

## Discussion

This case underscores a diagnostic challenge in sports cardiology: Following PE recovery, resumption of training precipitated rapid, persistent RV-predominant dilation co-occurring with a blunted chronotropic response, exercise-induced PVCs, and below-expected VO₂ for elite training status.^2^

The patient met no major 2010 or 2023 Task Force Criteria for arrhythmogenic RV cardiomyopathy (ARVC).^3,4^ CMR showed preserved biventricular function without LGE or wall motion abnormalities. ECG showed QRS fragmentation in V1, which could represent a sign of athlete’s heart reflecting dilation of the RV outflow tract particularly when persisting after deep inspiration.^5^ Exercise-induced PVCs, while meeting a minor criterion, displayed an infundibular pattern that can be benign in athletic hearts.^6^ The DSC2 variant remains of uncertain significance. Notably, symptoms evolved over months, unlike ARVC’s indolent progression to overt RV dysfunction.^7^

Exercise-induced ARVC (EIARVC) was considered but would typically involve RV dysfunction preceding dilation and fibrosis or scarring on CMR,^7^ which was not the case for our athlete. Nonetheless, QRS fragmentation and infundibular exercise PVCs—often benign but may carry greater clinical weight with RV enlargement—suggest subtle electrical perturbations that, with disproportionate RV-to-LV remodelling, place him in a clinical grey zone where extreme exercise may unmask subclinical susceptibility.^7,8^ This aligns with observations that endurance sports can exacerbate arrhythmogenic substrates in predisposed individuals,^9^ even without formal ARVC criteria. Pending validated diagnostic frameworks, such cases warrant ARVC exclusion per Task Force Criteria,^3,4^ documenting exercise-dose dependency,^7,10^ and surveillance for EIARVC phenotypes.^7^

Residual pulmonary vascular sequelae from prior PE were excluded: V/Q scintigraphy and V/Q SPECT ruled out perfusion mismatch or chronic thromboembolic disease. CPET showed normal ventilatory efficiency (VE/VCO₂ slope 23.2), preserved gas exchange (peak PETCO₂ 38 mmHg, no desaturation), and normal spirometry, arguing against post-PE pulmonary vasoconstriction or vasodilation as the primary aetiology.^11^ Transient exercise-induced bronchoconstriction was considered,^11,12^ but its role in persistent RV remodelling is uncertain, and provocation testing was deferred given symptom improvement.

The temporal dissociation between PE recovery and RV dilation implicates exercise-related haemodynamic stress as the primary driver of remodelling, with prior PE and possible exercise-induced bronchoconstriction as secondary contributors. The absence of residual clot burden favours exercise predominance, supported by marked chronotropic incompetence despite maximal effort (70% of age-predicted peak heart rate; RER 1.21). Peak VO₂, although 115% predicted (vs. expected ≥150% in endurance athletes^13^), likely reflects a decline from his elite baseline and is sustained through supranormal stroke volume (O₂ pulse 156% predicted), indicating cardiopulmonary impairment despite normal resting haemodynamics.^8^ Together, these findings blur the boundary between adaptation and maladaptation, suggesting a transitional state in which extreme exercise unmasks subclinical vulnerability.^2,7,8,10^

Monitored detraining provided both diagnostic and therapeutic insight. We engaged in shared decision-making with the athlete, carefully adjusting activity to reduce stress on the RV while respecting his training goals. Without high-risk features such as malignant arrhythmias or overt systolic dysfunction, an implantable cardioverter-defibrillator was not indicated. Nonetheless, despite symptomatic improvement, his RV remained markedly enlarged with persistent evidence of remodelling, highlighting ongoing vulnerability and the need for longitudinal surveillance to monitor potential progression or late arrhythmic risk.^5,7^

This is the first report of disproportionate RV remodelling precipitated by extreme training in a susceptible post-PE athlete and highlights the interplay between extreme physiology and potential pathology in athletes, exposing gaps in evaluating ultra-endurance athletes with borderline findings. Future studies using stress CMR or invasive CPET,^14,15^ alongside athlete registries, should determine whether these phenotypes reflect extreme adaptation, evolving pathology, or a distinct exercise-induced entity. Clinically, a nuanced approach integrating functional testing and advanced imaging is warranted, with vigilance for subtle maladaptation in high-intensity athletes.

## Lead author biography


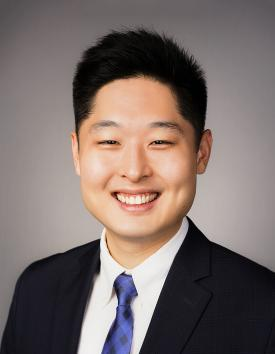


Dr. Jonathan S. Ahn graduated from California University of Science and Medicine in 2024. Following his graduation, he started his internal medicine residency at Loma Linda University Health, where he is currently a second-year resident. His professional interests within cardiology include sports cardiology, advanced heart failure, and electrophysiology.

## Supplementary Material

ytaf598_Supplementary_Data

## Data Availability

The data underlying this article will be shared on reasonable request to the corresponding author.

## Notes

**Funding:** The authors received no external funding for the conduct of this study.
